# Efficacy of chest X‐rays after drain removal in adult and pediatric patients undergoing cardiac and thoracic surgery: A systematic review

**DOI:** 10.1111/jocs.17114

**Published:** 2022-11-06

**Authors:** Myat S. Thet, Khin P. P. Han, Khun E. Hlwar, Khaing S. Thet, Aung Y. Oo

**Affiliations:** ^1^ Department of Surgery & Cancer, Faculty of Medicine Imperial College London London UK; ^2^ Mandalay General Hospital University of Medicine Mandalay Myanmar; ^3^ Department of Cardiothoracic Surgery St Bartholomew's Hospital London UK

**Keywords:** cardiac surgery, chest drain, chest tube, chest X‐ray, thoracic surgery

## Abstract

**Background:**

Chest X‐rays are routinely obtained after the removal of chest drains in patients undergoing cardiac and thoracic surgical procedures. However, a lack of guidelines and evidence could question the practice. Routine chest X‐rays increase exposure to ionizing radiation, increase health‐care costs, and lead to overutilisation of available resources. This review aims to explore the evidence in the literature regarding the routine use of chest X‐rays following the removal of chest drains.

**Materials & Method:**

A systematic literature search was conducted in PubMed, Medline via Ovid, Cochrane central register of control trials (CENTRAL), and ClinicalTrials. gov without any limit on the publication year. The references of the included studies are manually screened to identify potentially eligible studies.

**Results:**

A total of 375 studies were retrieved through the search and 18 studies were included in the review. Incidence of pneumothorax remains less than 10% across adult cardiac, and pediatric cardiac and thoracic surgical populations. The incidence may be as high as 50% in adult thoracic surgical patients. However, the reintervention rate remains less than 2% across the populations. Development of respiratory and cardiovascular symptoms can adequately guide for a chest X‐ray following the drain removal. As an alternative, bedside ultrasound can be used to detect pneumothorax in the thorax after the removal of a chest drain without the need for ionizing radiation.

**Conclusion:**

A routine chest X‐ray following chest drain removal in adult and pediatric patients undergoing cardiac and thoracic surgery is not necessary. It can be omitted without compromising patient safety. Obtaining a chest X‐ray should be clinically guided. Alternatively, bedside ultrasound can be used for the same purpose without the need for radiation exposure.

## 1 INTRODUCTION

Chest drains are widely used in cardiothoracic surgery. They are left in place after surgery for evacuation of air and fluids accumulated in the thoracic cavities. It has been a standard practice to obtain a routine chest X‐ray once the chest drain is removed. There is no clear guidance regarding the necessity and timing of the chest X‐rays after removal, yet they are rather empirically obtained.^1^, ^2^, ^3^ Americal College of Radiology does not support the routine use of chest X‐rays after the removal of chest drains.^4^

Undifferentiated routine use of chest X‐rays has several disadvantages. It increases false‐positive rates, overall costs, and leads to overutilisation of the health‐care resources.^5^, ^6^ Furthermore, it increases the exposure to ironizing radiation in patients, especially important in children because of the cumulative risk of malignancy later in life.^3^, ^7^ In addition, routine use of chest X‐rays does not influence mortality rate, length of intensive care stay, or hospital stay.^5^ This review aims to explore the evidence in the literature regarding the routine use of chest X‐rays in patients, both adults and children, undergoing cardiac and thoracic surgical procedures.

## 2 MATERIALS & METHODS

This systematic review follows the 2020 updated Preferred Reporting Items for Systematic Reviews and Meta‐Analyses (PRISMA) guideline.^8^

### 2.1 Literature search

We conducted a systematic literature search of studies investigating the effectiveness of routine chest X‐rays after drain removal in patients undergoing cardiothoracic surgery in Pubmed, Medline via Ovid, Cochrane central register of control trials (CENTRAL), and ClinicalTrials. gov. There was no limit on the publication year of the studies, and the last search was performed on April 17, 2022. The keywords used in combination with Boolean operators include “cardiac surgery,” “thoracic surgery,” “cardiothoracic surgery,” “chest drain removal,” “chest tube removal,” “chest X‐ray,” and “chest radiograph.” The references of the included studies and relevant reviews are also manually screened for identifying potentially eligible studies.

### 2.2 Inclusion and exclusion criteria

Original research studies reporting the incidence of pneumothorax and chest X‐ray findings after the chest drain removal in both adult and pediatric patients undergoing cardiac and thoracic surgery procedures were included. The exclusion criteria included case reports, case series, reviews, and studies on chest X‐rays after drain removal on the trauma patients exclusively.

### 2.3 Study screening, data extraction, and outcome measures

Two authors (K. P. P. H. and K. E. H.) independently reviewed the included studies and performed data extraction. Disagreements between the authors were resolved by consensus or by escalating to another author (M. S. T.). The following set of data was extracted: (i) study characteristics including study design, (ii) patient population characteristics, (iii) incidence of pneumothorax after removal of drain, (iv) clinical signs and symptoms following the removal of chest drain, and (v) reintervention (reinsertion) of chest drain. The primary outcome measure is the incidence of pneumothorax, whereas, the secondary outcomes include the need for reintervention following the removal of the chest drain, and signs and symptoms after chest drain removal.

### 2.4 Quality assessment

The quality of the included studies was independently assessed by two authors (K. P. P. H. and K. S. T.) using the Methodological Index for Nonrandomized Studies (MINORS).^9^ All included studies are moderate‐quality studies with a global score ranging from 8 to 12 out of 16.

## 3 RESULTS

**Figure 1:**
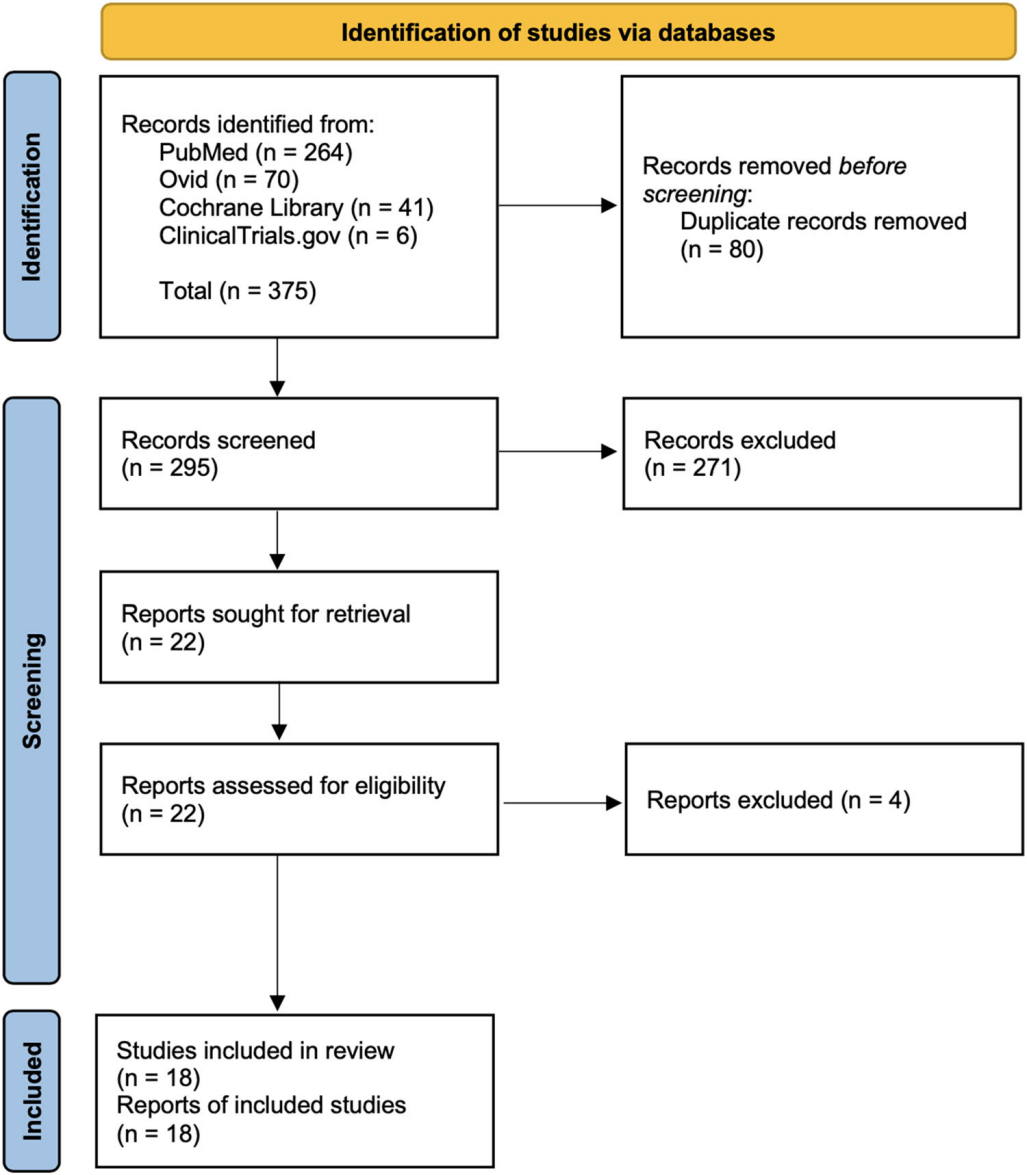
Preferred Reporting Items for Systematic Reviews and Meta‐Analyses (PRISMA) Flow Diagram

A total of 375 studies were retrieved through our search, and 80 duplicates were removed. The titles and abstracts of the remaining 295 studies were screened to identify potentially eligible 22 studies. After the full‐text screening, 18 studies were included in the systematic review. Eleven studies were conducted in the United States of America,^1^, ^2^, ^3^, ^7^, ^10^, ^11^, ^12^, ^13^, ^14^, ^15^, ^16^ four studies in the United Kingdom,^17^, ^18^, ^19^, ^20^ one in the Netherlands,^5^ one in Germany,^21^ and one in Iran^6^ (Table 1). No other studies were identified through manual screening of references of the included studies and relevant reviews. PRISMA flow diagram is demonstrated in Figure 1. Adult patients refer to patients at least 18 years of age, and pediatric patients refer to patients under the age of 18 in the studies. Reintervention is defined as the reinsertion of a chest drain.

**Table 1:** Study characteristics included in the systematic review

Author	Year	Study design	Patient population	Number of patients	Country
Pacharn et al.	2001	Retrospective cohort	Cardiac surgery, pediatric	374	United States
McCormick et al.	2002	Retrospective cohort	Cardiac surgery, adult	1000	United Kingdom
Mandegar et al.	2007	Randomized controlled trial	Cardiac surgery, adult	315	Iran
Khan et al.	2008	Prospective cohort	Cardiac surgery, adult	151	United Kingdom
Whitehouse et al.	2009	Prospective cohort	Thoracic surgery, adult	74	United Kingdom
Stather et al.	2010	Prospective cohort	Cardiac and thoracic surgery, pediatric	93	United Kingdom
Saucier et al.	2010	Case control	Thoracic surgery, adult	50	United States
Eisenberg et al.	2010	Prospective cohort	Cardiac surgery, adult	400	United States
Tolsma et al.	2011	Prospective cohort	Cardiac surgery, adult	214	The Netherlands
Woodward et al.	2011	Prospective cohort	Cardiac surgery, pediatric	53	United States
Cunningham et al.	2014	Retrospective cohort	Thoracic surgery, pediatric	146	United States
Johnson et al.	2017	Retrospective cohort	Thoracic surgery, pediatric	179	United States
McGrath et al.	2017	Retrospective cohort	Thoracic surgery, pediatric	281	United States
Galetin et al.	2019	Prospective cohort	Thoracic surgery, adult	123	Germany
Porter et al.	2019	Retrospective cohort	Thoracic surgery, adult	241	United States
LaGrasta et al.	2020	Retrospective cohort	Cardiac surgery, pediatric	11,651	United States
Kanamori et al.	2021	Retrospective cohort	Thoracic surgery, pediatric	102	United States
Zukowski et al.	2022	Retrospective cohort	Thoracic surgery, adult	433	United States

### 3.1 Cardiac surgery adult patients

One simple randomized trial,^6^ three prospective cohort studies,^5^, ^11^, ^19^ and one retrospective cohort study^18^ evaluated the efficacy of routine chest X‐rays constituting a total of 2080 adult patients undergoing cardiac surgery procedures, which includes coronary artery bypass grafting (CABG), valve surgery, and atrial septal defect repair, either as isolated procedures or in combination. The mean duration of chest drain before removal is 1.4–1.8 days,^11^, ^18^, ^19^ and a routine chest X‐ray is obtained within 3–4 h after the removal.^6^, ^19^

If a chest X‐ray is obtained after the removal, the incidence of pneumothorax ranges from 1.3% to 9.3% with up to 1.3% of patients developing respiratory symptoms or hemodynamic changes and reintervention was required in 0.5%–1.3% of the patients.^5^, ^6^, ^11^, ^18^, ^19^ If no routine chest X‐ray was performed after the drain removal, 5.0%–11.8% of the patients developed respiratory symptoms or hemodynamic changes, of which 0.7%–2.5% would require reintervention.^6^, ^18^

### 3.2 Cardiac surgery pediatric patients

Two prospective cohort studies^10^, ^20^ and two retrospective cohort studies^7^, ^14^ have examined the incidence of pneumothorax and the efficacy of chest X‐rays after the chest drain removal. It includes a total of 12,171 pediatric patients undergoing congenital cardiac surgery operations. The mean age of the patients ranges from 0.2 to 2.7 years. The mean duration of chest drain varies from 1 to 6.4 days after the procedure, and a chest X‐ray is performed within 2–6 h after the removal of the drain.^7^, ^10^, ^14^, ^20^

The incidence of pneumothorax after chest drain removal is 0.21% in a study with 11,651 patients^7^ and goes as high as 13.6% in other studies.^10^, ^14^, ^20^ Clinical symptoms developed in 0.1%–1.87% of the patients, and 0.1%–1.6% of patients would eventually undergo reintervention for the pneumothorax.^7^, ^14^

### 3.3 Thoracic surgery adult patients

Three prospective studies^15^, ^17^, ^21^ and two retrospective studies^1^, ^2^ including 921 patients explored the effectiveness of routine chest X‐rays in adult patients undergoing thoracic surgical procedures, which include both video‐assisted thoracoscopy (VATS) and open surgery. The patients have a mean age of 60–64 years in the studies and had the chest drains for an average duration ranging from 1 to 4 days. A chest X‐ray is usually performed 2–4 h after the removal of the chest drain.^1^

The incidence of pneumothorax on the chest X‐ray after removal of the drain varies greatly from 4% to 48%.^1^, ^2^, ^15^, ^17^, ^21^ Nonetheless, only up to 0.5% of patients need a subsequent intervention after the positive radiographic findings.^1^, ^2^, ^17^ The incidence of pneumothorax in asymptomatic patients is approximately 32.8%, but none of the patients required reintervention. They only had clinical observation and repeated the chest X‐rays.^1^

### 3.4 Thoracic surgery pediatric patients

Four retrospective studies with a total of 708 pediatric patients investigated the necessity of performing routine chest X‐rays after the removal of chest drains.^3^, ^12^, ^13^, ^16^ The mean age of patients in the studies ranges from 7.5 to 9.4 years. The patients underwent various thoracic surgical procedures including VATS and open surgeries. The average length of the chest drain is 3.7–7.2 days before removal, and the chest X‐rays are obtained within 2–6 h after the removal of the drain.

In the postdrain removal chest X‐rays, the incidence of pneumothorax is 3.1%–3.9%^3^, ^12^ with a reintervention rate is 0.7%–1.7%.^12^, ^13^, ^16^ Clinical symptoms were present in all patients who required reintervention. In asymptomatic patients, the incidence of pneumothorax is 1.8%,^13^ of which 0.4%–0.9% of the asymptomatic patients would undergo further reintervention.^13^, ^16^

### 3.5 Bedside ultrasound versus chest X‐ray after removal of chest drain

There are two prospective observational studies with combined 173 patients, which evaluated the role of bedside ultrasound in comparison to using chest X‐ray after removal of a chest drain to detect pneumothorax, predominantly in the thoracic adult surgical population.^15^, ^21^ The overall sensitivity of the bedside ultrasound is 32%, however, the sensitivity is increased to 100% in detecting pneumothoraces of 3 cm or larger, and the specificity is 85%.^21^ There is a strong association between ultrasound and chest X‐ray with a therapeutic agreement of 97%.^21^ There is perfect agreement between the two methods with a **κ** statistics value of 1.000.^15^

## 4 DISCUSSION

Studies in the literature recommend that there is no need to routinely perform a chest X‐ray after the removal of a chest drain following cardiac or thoracic surgery. Diverse patient populations undergoing different cardiac and thoracic surgeries have demonstrated the incidence of pneumothorax that requires intervention after the removal of chest drain is low and it is highly correlated with the development of clinical symptoms. Approximately 1–2% of patients would require reintervention after the chest drain removal, with the exception of the adult thoracic surgical population, which may be up to 10% of the patients. The high incidence could possibly be contributed by the pre‐existing pulmonary pathology and complex physiological changes within the pleural space associated with the thoracic surgical procedures.^1^

It was previously thought the pediatric population might develop a higher rate of pneumothorax occurrence since young children are unlikely to be able to follow breathing commands during the chest drain removal.^20^ However, it is not the case when a large study with more than 11,000 pediatric patients in cardiac surgery demonstrated the incidence of pneumothorax as less than 1%.^7^ Nevertheless, a good removal technique is essential to reduce the rate of complications.^20^

Current literature agrees that performing a chest X‐ray is only necessary if a patient develops respiratory or cardiovascular symptoms after the removal of the chest drain given that most clinically significant pneumothorax will eventually develop clinical symptoms. Omitting a chest X‐ray will significantly reduce the health‐care cost,^1^, ^12^, ^14^, ^18^, ^19^ as well as radiation exposure in pediatric patients.^12^ Moreover, routine chest X‐rays lead to additional subsequent chest X‐rays without the further need for intervention.^2^, ^14^, ^18^

On the other hand, a clinically significant pneumothorax might develop after the removal of the chest drain despite the lack of symptoms. Especially in very young patients, who are not able to communicate, it could be concerning that a moderate or large pneumothorax may not exhibit clinical symptoms in the context of a lack of good respiratory or cardiovascular physiological reserve, despite very few of them requiring subsequent intervention.^7^ Assessment of clinical symptoms solely might not be sufficient, and subgroups of patients with risk factors should be identified to acquire chest X‐rays following the removal of the chest drain.^5^, ^7^ Nonetheless, a greater majority of studies reinforce the fact that routine use of chest X‐rays can be eliminated without compromising patient safety.

An alternative to obtaining a chest X‐ray after the removal of a drain is bedside ultrasound. Despite overall low sensitivity and specificity compared with X‐rays, the bedside ultrasound is highly accurate in identifying a clinically significant pneumothorax.^21^ It is safe, has no ionizing radiation, has lower cost compared with X‐rays, and has rapid instant interpretation compared with ordering, obtaining, and interpreting a chest X‐ray. It is reproducible and requires minimum experience to detect pneumothorax, with the benefit of detecting further diagnoses such as pleural effusion and pericardial effusion.^15^ Yet, the use of ultrasound could be limited by subjective interpretation of findings depending on the experience of the operator, postoperative surgical dressings, and anatomical and physiological changes, especially after thoracic surgery. In spite of the limitations, an overall low incidence of pneumothorax after chest drain removal would further favor the use of bedside ultrasound instead of chest X‐rays.

## 5 CONCLUSION

A routine chest X‐ray following chest drain removal in adult and pediatric patients undergoing cardiac and thoracic surgery is not necessary. It can be omitted without compromising patient safety. Obtaining chest X‐rays should be guided by the development of respiratory and cardiovascular symptoms. An alternative to the chest X‐ray is to perform a bedside ultrasound, which is rapid, safe, cost‐effective, and accurately identifies pneumothorax without ionizing radiation. Further research is required to identify the asymptomatic patients with a clinically significant pneumothorax, who may need subsequent intervention.
